# The association between gestational hypothyroidism in pregnant women with preeclampsia, maternal liver function indicators, and neonatal birth weight: a study in Chinese pregnant women

**DOI:** 10.3389/fendo.2025.1555277

**Published:** 2025-09-22

**Authors:** Fang Zhang, Qing Hua, Xiaoyan You, Fenglian Shi, Yadan Zhou, Xia Xu, Xiaona Tian, Gang Tian, Li Li

**Affiliations:** ^1^ Department of Obstetrics, Zhengzhou Central Hospital Affiliated to Zhengzhou University, Zhengzhou, China; ^2^ Institute of Trauma and Metabolism, Zhengzhou Central Hospital Affiliated to Zhengzhou University, Zhengzhou, China; ^3^ Henan Province Hypertension Precision Prevention and Control Engineering Research Center, Henan Provincial People’s Hospital, Zhengzhou University People’s Hospital, Zhengzhou, Henan, China

**Keywords:** birth weight, preeclampsia, gestational hypothyroidism, liver function, mediating model

## Abstract

Birth weight serves as a critical indicator of neonatal survival. Preeclampsia represents a serious complication during pregnancy and is closely associated with gestational hypothyroidism (GHT), both of which severely affect neonatal birth weight. Preeclampsia and hypothyroidism during pregnancy are usually accompanied by abnormalities of maternal liver function, which frequently leads to adverse pregnancy outcomes including low birth weight (LBW). This retrospective study utilized data from 420 cases of patients with preeclampsia who underwent prenatal examinations and delivery at department of Obstetrics. The association between preeclampsia combined with GHT in pregnancy, maternal liver function and neonatal birth weight was estimated using generalized linear model (GLM), and the potential partial mediating effects of maternal liver function were assessed through mediating models. Among pregnant women with preeclampsia, 11.0% had GHT, and the median (interquartile range) birth weight of all neonates was 2990.0 (2541.3, 3368.8) grams. Neonates born to pregnant women who had preeclampsia combine with GHT showed a higher incidence of LBW (*χ*²=22.13, *P*< 0.001), exhibited a significantly lower birth weight compared to those born to women with preeclampsia alone (*β*=-258.53;95%*CI*:-398.56, -118.50). Additionally, maternal alanine aminotransferase (ALT) levels were found to partially mediate this association (indirect effect:-50.85, 95%*CI*:-101.07, -15.07). The findings of this study indicate that compared with pregnant women with preeclampsia alone, neonates born to pregnant women suffering from preeclampsia combined with GHT have significantly lower birth weights, with maternal ALT levels acting as a potential partial mediator in this association. These results provide an important reference for clinicians to monitor thyroid and liver function in patients with preeclampsia.

## Introduction

Neonatal birth weight is determined by maternal health, the placenta, and the fetus’ own growth potential, and serving as a critical indicator of neonatal survival (1, 2). Low birth weight (LBW), defined as a birth weight of less than 2500 grams(g), is considered an important factor in neonatal mortality (3, 4). Studies have demonstrated that LBW increases the risk of future cardiovascular morbidity and is associated with an elevated risk of future hypertension in pregnancy (5, 6).

Preeclampsia is a serious complication of pregnancy with hypertension and proteinuria as the main clinical manifestations, and is one of the leading causes of maternal and neonatal mortality (7, 8). Preeclampsia can cause a series of serious obstetric complications, including preterm labor and placental abruption, as well as fetal complications such as fetal respiratory distress, intrauterine growth restriction, oligohydramnios, and stillbirth (9). There is increasing evidence suggests that preeclampsia is closely associated with maternal hypertension, cardiovascular disease, and dementia (10–12). Mechanistically, placental dysfunction induced by preeclampsia profoundly impacts on fetal development, with studies confirming it as an important predictor of neonatal birth weigh (13–15). The thyroid gland is involved in endocrine regulation and plays a crucial role in maternal and fetal development during pregnancy (16). Studies have confirmed the correlation between thyroid dysfunction and preeclampsia, and the prevalence of hypothyroidism in pregnant women with preeclampsia is significantly increased (17–19). Currently, hypothyroidism has become a common complication of preeclampsia, leading to adverse pregnancy outcomes, including LBW, and severely affecting neonatal birth weight and even future growth and development (20).

Preeclampsia and hypothyroidism during pregnancy are closely associated with alterations in liver function. Preeclampsia causes impaired liver function, which has been identified as the third most important predictor after hypertension and proteinuria (21, 22). Simultaneously, hypothyroidism, which is characterized by a feedback increase in thyroid-stimulating hormone (TSH) as a biochemical marker, interacts with hepatic function metabolically (23, 24). Experimental evidence has been presented that hepatic dysfunction in pregnant mice predisposes to placental dysfunction, which results in lower birth weights in newborn mice (25). Population-based studies have also confirmed that pregnant women with abnormal liver function are associated with adverse birth outcomes, such as preterm labor, LBW, intrauterine stillbirth, and fetal respiratory distress (26). Several scholars have investigated the mechanisms underlying the association of preeclampsia and hypothyroidism in pregnancy with LBW, including placental dysfunction in preeclampsia, maternal nutritional deficiencies associated with pregnancy, and maternal thyroid hormone levels (27–29). In conclusion, the mechanism by which preeclampsia combined with hypothyroidism affects neonatal birth weight is multifactorial. But there are currently limited studies exploring the mediating role of liver function as an important factor in the relationship between preeclampsia combined with hypothyroidism and birth weight. As shown in some studies, maternal liver function status is associated with fetal growth and development during pregnancy. Conducting research on the association between liver function indicators and birth weight can provide a basis for early risk monitoring strategies.

Therefore, the objective of this study was to explore the association between preeclampsia in conjunction with gestational hypothyroidism, maternal liver function, and neonatal birth weight. Additionally, the study aimed to explore whether maternal liver function serves as a potential mediating factor in the association between preeclampsia combined with gestational hypothyroidism and neonatal birth weight. This investigation seeks to address existing gaps in the literature regarding the underlying mechanisms and to offer insights for future related studies.

## Materials and methods

### Study design and population

This study was a retrospective study, and 554 cases who underwent prenatal examinations and deliveries at department of Obstetrics, Zhengzhou Central Hospital Affiliated to Zhengzhou from January 2021 to September 2023 were selected as the study subjects. According to the following inclusion criteria, 482 cases diagnosed with preeclampsia were initially included, and then 62 cases were excluded according to the exclusion criteria, 420 cases of preeclampsia patients were ultimately included in this study, and the median gestational age at diagnosis of preeclampsia (interquartile range) was 35.4(32.5, 37.3) weeks. Among these, 46 cases were complicated by hypothyroidism, and the median gestational age at diagnosis of hypothyroidism (interquartile range) was 32.2(27.0, 36.0) weeks.

The inclusion criteria were as follows: (1) fulfillment of the diagnostic criteria for preeclampsia; (2) maternal age ≥ 18 years; (3) gestational age ≥ 24 weeks; (4) singleton pregnancy; (5) absence of a history of substance abuse, smoking, or alcohol consumption among the pregnant women.

The exclusion criteria were as follows: (1) pregnant individuals with other complications, such as gestational diabetes or hypertension, as well as those with underlying medical conditions prior to pregnancy (e.g., thyroid disorders, chronic hypertension, heart disease, liver or biliary diseases, or renal diseases); (2) patients lacking relevant information, such as those with incomplete or missing neonatal birth weight and liver function indicators; (3) individuals who conceived through assisted reproductive technology.

The diagnostic criteria for preeclampsia were based on the “Diagnosis and treatment of hypertension and preeclampsia in pregnancy: a clinical practice guideline in China (2020)” issued by the Obstetrics and Gynecology Branch of the Chinese Medical Association. Specifically, preeclampsia is diagnosed when, after 20 weeks of gestation, a pregnant woman exhibits a systolic blood pressure of ≥140 mmHg and/or a diastolic blood pressure of ≥90 mmHg, accompanied by at least one of the following: (1) a 24-hour urinary protein quantification of ≥0.3 g, or a urinary protein-to-creatinine ratio of ≥0.3, or a random urinary protein result of ≥(+); (2) the absence of proteinuria but the presence of any one of the following organ or system involvements, including abnormalities affecting vital organs such as the heart, lungs, liver, and kidneys, or alterations in the hematological, gastrointestinal, or neurological systems, as well as involvement of the placenta and fetus (30).

The diagnostic criteria for hypothyroidism during pregnancy refer to the revised “Guideline on diagnosis and management of thyroid diseases during pregnancy and postpartum (2^nd^ edition)” by the Chinese Medical Association, which stipulates that serum thyroid-stimulating hormone (TSH) levels exceed the upper limit of the pregnancy-specific reference range, while serum free thyroxine (FT4) levels fall below the lower limit of the specific reference range. Combined with the types of kits and fully automated chemiluminescent immunoassay analyzers used in this study, the guideline recommended reference ranges for TSH and FT4 were as follows: in early pregnancy, TSH 0.05~3.55 mIU/L, FT49.01~15.89 pmol/L; in mid-pregnancy, TSH 0.21~3.31 mIU/L, FT4 6.62~13.51 pmol/L; in late pregnancy, TSH 0.43~3.71 mIU/L, FT4 6.42~10.75 pmol/L (31).

### Ethical compliance statement for human participant research

All study participants provided written informed consent, and this study received approved from the Ethics Committee of Zhengzhou Central Hospital Affiliated to Zhengzhou (No. ZXYY202470). All methods were performed in accordance with the relevant guidelines and regulations of the Declaration of Helsinki.

### Variables and definitions

#### Measurement of serum liver function indicators

In this study, we collected the levels of liver function indicators from subjects during their hospitalization. Based on previous research, this study selected indicators closely related to liver function, primarily including alanine aminotransferase (ALT 7-40U/L), aspartate aminotransferase (AST 13-35U/L), alkaline phosphatase (ALP 50-135U/L), total bilirubin (TBIL 0-21μmol/L), total protein (TP 60-80g/L), and albumin (Alb 35-55g/L). A volume of 5 mL of fasting antecubital venous blood was collected, and serum was separated by centrifugation at 4000 rpm for 5 minutes. All biochemical analyses were performed using an AU5800 fully automated biochemistry analyzer (Beckman Coulter, USA) with matched reagent kits. All reagents and instruments were subjected to quality control procedures.

#### Measurement of neonatal birth weight

Neonatal birth weight, measured in grams, was measured and recorded within one hour after birth. The data pertaining to birth weight for this investigation was sourced from medical records.

#### Measurement of covariates

Participants in this study were requested to complete a baseline questionnaire upon admission, which encompassed demographic characteristics of the pregnant women (age, ethnicity, residence, and educational level), history of cesarean delivery (yes/no), history of adverse pregnancy outcomes (yes/no), primiparity (yes/no), and family history of hypertension (yes/no). Participants self-reported their pre-pregnancy weight (kg) and their height was measured in a barefoot standing position using a medical height and weight measuring device (cm). Subsequently, pre-pregnancy body mass index (PBMI) was calculated using the standard formula BMI = weight (kg)/height (m²). According to World Health Organization (WHO) standards, participants were classified into categories of underweight (BMI< 18.5 kg/m²), normal weight (18.5 ≤ BMI ≤ 24.9 kg/m²), overweight (25 ≤ BMI ≤ 29.9 kg/m²), and obese (BMI ≥ 30 kg/m²). During hospitalization, ultrasound was utilized to assess whether the fetus was experiencing growth restriction, and postpartum data on preterm birth (yes/no) and neonatal sex (male/female) were collected from medical records. The ultrasound diagnostic criteria for fetal growth restriction (FGR) were defined as an ultrasound-estimated fetal weight or abdominal circumference below the 10th percentile for the corresponding gestational age; preterm birth was defined as delivery occurring before 37 weeks of gestation.

### Statistical analysis

Data analysis was conducted using SPSS 26.0 statistical software. Initially, descriptive analysis was performed, with categorical data expressed as N (%) and continuous data described using either $\bar{x}\pm s$ or M (IQR). For univariate analysis, given that birth weight exhibited a non-normal distribution, the Mann-Whitney U test or Kruskal-Wallis H test were employed to examine differences in birth weight among various characteristic groups. Spearman correlation analysis was utilized to assess the relationship between liver function indicators (ALT/AST/ALP/TP/Alb/TBIL) and birth weight. Subsequently, a generalized linear model (GLM) was applied for multivariate analysis to evaluate the potential association between the presence of GHT, the levels of liver function indicators, and birth weight. Finally, the presence of GHT was treated as the independent variable, liver function indicators as mediating variables, and birth weight as the dependent variable. The mediation effect of liver function indicators was calculated using the SPSS Process macro, employing the bias-corrected Bootstrap method (with 5000 resamples) for validation. A p-value of<0.05 was considered statistically significant.

## Results

### Descriptive statistics

This study included a total of 420 pregnant women diagnosed with preeclampsia, among whom 46 (11.0%) also had concomitant hypothyroidism. The median age (interquartile range) was 31 (28, 34) years, with the majority of the participants (72.1%) aged between 25 and 35 years. The median neonatal birth weight (interquartile range) was 2990.0 (2541.3, 3368.8) grams. Preterm birth occurred in 111 participants (26.4%), while 24 (5.7%) were diagnosed with fetal growth restriction. Additionally, 15 participants were classified as underweight prior to pregnancy, 123 as overweight, and 52 as obese. Univariate analysis revealed that preterm birth (*P*<0.001), occurrence of fetal growth restriction during pregnancy (*P*<0.001), a pre-pregnancy BMI below 18.5 kg/m² (*P*=0.003), and the presence of GHT (*P*<0.001) were associated with lower birth weights of the neonates born to women with preeclampsia (**Table 1**).

**Table 1:** The baseline characteristics of the included pregnant women.

Variable	N (%)	Birth weight (g)	*Z*/*H*	*P*
Age (Years)				
<25	23 (5.5)	2980.0 (2610.0, 3250.0)	2.65	0.449
25~35	303 (72.1)	3000.0 (2600.0, 3370.0)		
35~40	79 (18.8)	2825.0 (2325.0, 3350.0)		
≥40	15 (3.6)	3155.0 (2795.0, 3630.0)		
Fetal sex				
Male	223 (53.1)	3015.0 (2600.0, 3370.0)	-0.68	0.494
Female	197 (46.9)	2950.0 (2497.5, 3365.0)		
Ethnicity				
Han ethnicity	411 (97.9)	2990.0 (2550.0, 3365.0)	-0.65	0.513
Ethnic minorities	9 (2.1)	2890.0 (2120.0, 3485.0)		
Residence				
Urban area	349 (83.1)	2980.0 (2547.5, 3322.5)	-1.26	0.208
Rural area	71 (16.9)	3100.0 (2500.0, 3570.0)		
Education level				
Junior high school and below	41 (9.8)	3155.0 (2260.0, 3500.0)	2.11	0.550
High school and vocational secondary school	51 (12.1)	2980.0 (2450.0, 3305.0)		
Junior college	134 (31.9)	3032.5 (2660.0, 3415.0)		
Undergraduate and postgraduate degrees	194 (46.2)	2950.0 (2543.8, 3311.3)		
PTD				
No	309 (73.6)	3160.0 (2880.0, 3495.0)	-12.79	<0.001
Yes	111 (26.4)	2240.0 (1985.0, 2525.0)		
History of cesarean section				
No	319 (76.0)	3005.0 (2545.0, 3370.0)	-0.68	0.496
Yes	101 (24.0)	2965.0 (2522.5, 3315.0)		
History of adverse obstetric				
No	363 (86.4)	3010.0 (2570.0, 3370.0)	-1.66	0.098
Yes	57 (13.6)	2760.0 (2450.0, 3275.0)		
Primipara				
No	167 (39.8)	2950.0 (2495.0, 3370.0)	-1.06	0.288
Yes	253 (60.2)	3020.0 (2570.0, 3367.5)		
FGR				
No	396 (94.3)	3017.5 (2630.0, 3370.0)	-5.92	<0.001
Yes	24 (5.7)	2182.5 (1788.8, 2338.8)		
Family history of hypertension				
No	370 (88.1)	3002.5 (2548.8, 3370.0)	-1.28	0.201
Yes	50 (11.9)	2895.0 (2498.8, 3242.5)		
PBMI (kg/m^2^)				
<18.5	15 (3.6)	2645.0 (2255.0, 3165.0)	13.77	0.003^*^
18.5~24.9	230 (54.7)	2950.0 (2392.5, 3280.0)		
25.0~29.9	123 (29.3)	3035.0 (2660.0, 3390.0)		
≥30	52 (12.4)	3180.0 (2808.8, 3613.8)		
PE&GHT				
No	374 (89.0)	3015.0 (2610.0, 3371.3)	-3.95	<0.001
Yes	46 (11.0)	2530.0 (2023.8, 3143.8)		

*P<0.05; PTD, Preterm delivery; FGR, Fetal growth restriction; PBMI, Pre-pregnancy body mass index; PE&GHT, Preeclampsia combined with gestational hypothyroidism.

Compared with pregnant women with preeclampsia alone, those with preeclampsia combined with GHT had higher rates of preterm delivery (39.1% vs. 24.9%), fetal growth restriction (17.4% vs. 4.3%) and LBW (50.0% vs. 19.3%), were more likely to be primiparous (73.9% vs. 58.6%), and obese (13.0% vs. 12.3%). From the perspective of liver function indicators, the levels of ALT (*P*<0.001) and AST (*P*=0.002) showed statistically significant differences between the two subgroups (**Table 2**).

**Table 2:** Comparison of baseline data between the preeclampsia group and the preeclampsia with hypothyroidism group.

Variable	Maternal status [n (%)/M (P_25_, P_75_)]		$\chi^{2}$ /*Z*	*P*
	PE&GHT	PE		
Age (Years)				
<25	0 (0.0)	23 (6.1)	-1.07	0.283
25~35	35 (76.1)	268 (71.7)		
35~40	7 (15.2)	72 (19.3)		
≥40	4 (8.7)	11 (2.9)		
Fetal sex				
Male	22 (47.8)	201 (53.7)	0.58	0.448
Female	24 (52.2)	173 (46.3)		
Ethnicity				
Han ethnicity	45 (97.8)	366 (97.9)	—	1.000
Ethnic minorities	1 (2.2)	8 (2.1)		
Residence				
Urban area	43 (93.5)	306 (81.8)	3.97	0.058
Rural area	3 (6.5)	68 (18.2)		
Education level				
Junior high school and below	6 (13.0)	35 (9.4)	-0.28	0.778
High school and vocational secondary school	6 (13.0)	45 (12.0)		
Junior college	10 (21.8)	124 (33.2)		
Undergraduate and postgraduate degrees	24 (52.2)	170 (45.4)		
PTD				
No	28 (60.9)	281 (75.1)	4.29	0.038^*^
Yes	18 (39.1)	93 (24.9)		
History of cesarean section				
No	37 (80.4)	282 (75.4)	0.57	0.451
Yes	9 (19.6)	92 (24.6)		
History of adverse obstetric				
No	39 (84.8)	324 (86.6)	0.12	0.730
Yes	7 (15.2)	50 (13.4)		
Primipara				
No	12 (26.1)	155 (41.4)	4.03	0.045^*^
Yes	34 (73.9)	219 (58.6)		
FGR				
No	38 (82.6)	358 (95.7)	10.75	0.001^*^
Yes	8 (17.4)	16 (4.3)		
Family history of hypertension				
No	43 (93.5)	327 (87.4)	1.43	0.232
Yes	3 (6.5)	47 (12.6)		
PBMI (kg/m^2^)				
<18.5	1 (2.2)	14 (3.7)	-2.33	0.020^*^
18.5~24.9	32 (69.6)	198 (53.0)		
25.0~29.9	7 (15.2)	116 (31.0)		
≥30	6 (13.0)	46 (12.3)		
Neonatal birth weight status				
non-LBW	23 (50.0)	302 (80.7)	22.13	<0.001
LBW	23 (50.0)	72 (19.3)		
ALT (U/L)	23.5 (12.8,59.3)	13.0 (9.0,20.0)	-4.34	<0.001
AST (U/L)	36.5 (24.0,50.3)	26.0 (22.0,33.0)	-3.17	0.002^*^
ALP (U/L)	155.0 (120.3,193.8)	151.0 (123.0,189.3)	-0.22	0.823
TP (g/L)	56.0 (52.4,62.0)	58.1 (54.0,62.0)	-1.18	0.238
Alb (g/L)	30.1 (27.5,34.4)	31.1 (29.0,34.0)	-1.66	0.097
TBIL (μmol/L)	10.9 (8.3,14.8)	11.0 (9.1,13.3)	-0.16	0.870

*P<0.05; PE&GHT, Preeclampsia combined with gestational hypothyroidism; PTD, Preterm delivery; FGR, Fetal growth restriction; PBMI, Pre-pregnancy body mass index; LBW, low birth weight; ALT, Alanine Aminotransferase; AST, Aspartate Aminotransferase; ALP, Alkaline Phosphatase; TP, Total Protein; Alb, Albumin; TBIL, Total Bilirubin; BW, Birth Weight.

### Correlation between maternal liver function indicators and neonatal birth weight

Except for total bilirubin, the levels of other maternal liver function indicators showed significant correlations with neonatal birth weight. Specifically, ALT (*r*=-0.320) and AST (*r*=-0.234) levels exhibited negative correlations with neonatal birth weight, while ALP (*r*=0.193), TP (*r*=0.165), and ALB (*r*=0.177) displayed positive correlations with neonatal birth weight (**Table 3**).

**Table 3:** Analysis of the correlation of maternal liver function indicators and neonatal birth weight.

Variables	ALT	AST	ALP	TP	ALB	TBIL	BW
ALT	1.000						
AST	0.589^**^	1.000					
ALP	0.111^*^	0.051	1.000				
TP	-0.038	-0.139^**^	0.077	1.000			
Alb	0.000	-0.181^**^	0.099^*^	0.790^**^	1.000		
TBIL	0.100^*^	0.118^*^	0.130^**^	0.067	0.127^**^	1.000	
BW	-0.320^**^	-0.234^**^	0.193^**^	0.165^**^	0.177^**^	0.089	1.000

* *P*<0.05, ** *P*<0.01. ALT, Alanine Aminotransferase; AST, Aspartate Aminotransferase; ALP, Alkaline Phosphatase; TP, Total Protein; Alb, Albumin; TBIL, Total Bilirubin; BW, Birth Weight.

### Relationship between thyroid function, liver function indicators in pregnant women with preeclampsia, and neonatal birth weight

This study explored the relationship between the presence of GHT in preeclamptic pregnant women and their liver function indicators with neonatal birth weight through the construction of a GLM and the adjustment of control variables. The findings revealed that, without adjusting for other variables (Model 1), the neonatal birth weight of infants born to preeclamptic mothers with GHT was significantly lower compared to those born to mothers with uncomplicated preeclampsia [*β* = -351.36; 95% confidence interval (*CI)*: -532.57, -170.15]. Furthermore, as levels of ALT (*β* = -2.18; 95% *CI*: -3.90, -0.47) and AST (*β* = -4.04; 95% *CI*: -7.06, 1.02) increased, neonatal birth weight correspondingly decreased, while an elevation in ALP levels (*β*=1.43; 95% *CI*: 0.53, 2.32) was associated with an increase in neonatal birth weight. Following adjustments for preterm birth, FGR, and PBMI (Model 2), the presence of GHT (*β* = -258.53; 95% *CI*: -398.56, -118.50), ALT (*β* = -1.88; 95% *CI*: -3.19, -0.56), and ALP (*β*=1.02; 95% *CI*: 0.33, 1.70) levels remained significantly associated with neonatal birth weight, whereas the association between AST levels (*β* = -1.34; 95% *CI*: -3.66, 0.98) and neonatal birth weight became statistically insignificant (**Table 4**).

**Table 4:** The results of generalized linear regression analyses of birth weight.

Variable	Model 1		Model 2	
	*β* (95%*CI*)	*P*	*β* (95%*CI)*	*P*
PE&GHT				
No (ref.)	—	—	—	—
Yes	-351.36 (-532.57, -170.15)	<0.001	-258.53 (-398.56, -118.50)	<0.001
ALT	-2.184 (-3.90, -0.47)	0.013^*^	-1.88 (-3.19, -0.56)	0.005^*^
AST	-4.04 (-7.06, 1.02)	0.009^*^	-1.34 (-3.66, 0.98)	0.256
ALP	1.43 (0.53, 2.32)	0.002^*^	1.02 (0.33, 1.70)	0.004^*^
TP	10.45 (-1.65, 22.56)	0.090	6.07 (-3.21, 15.34)	0.200
ALB	4.74 (-14.59, 24.07)	0.631	-0.45 (-15.24, 14.35)	0.953
PTD				
No (ref.)	—	—	—	—
Yes	—	—	-799.73 (-899.72, -699.75)	<0.001
FGR				
No (ref.)	—	—	—	—
Yes	—	—	-296.60 (-487.72, -105.49)	0.002^*^
PBMI (kg/m^2^)				
<18.5	—	—	-177.45 (-408.54, 53.64)	0.132
18.5~24.9 (ref.)	—	—	—	—
25.0~29.9	—	—	93.69 (-4.30, 191.68)	0.061
≥30.0	—	—	310.39 (176.83, 443.96)	<0.001

*P<0.05. CI, confidence interval; PE&GHT: Preeclampsia combined with gestational hypothyroidism; ALT, Alanine Aminotransferase; AST, Aspartate Aminotransferase; ALP, Alkaline Phosphatase; TP, Total Protein; Alb, Albumin; PTD, Preterm delivery; FGR, Fetal growth restriction; PBMI, Pre-pregnancy body mass index; ref., Reference Group.

Model 1 was the unadjusted model; Model 2 adjusted for PTD, FGR and PBMI.

We also did some extra analysis after sorting out liver function indicators (normal or abnormal) and birth weight (low birth weight or non-low birth weight). Check out the **Supplementary Table S1** and **Supplementary Table S2** for more details.

### Mediating effects of liver function indicators

**Figure 1:**
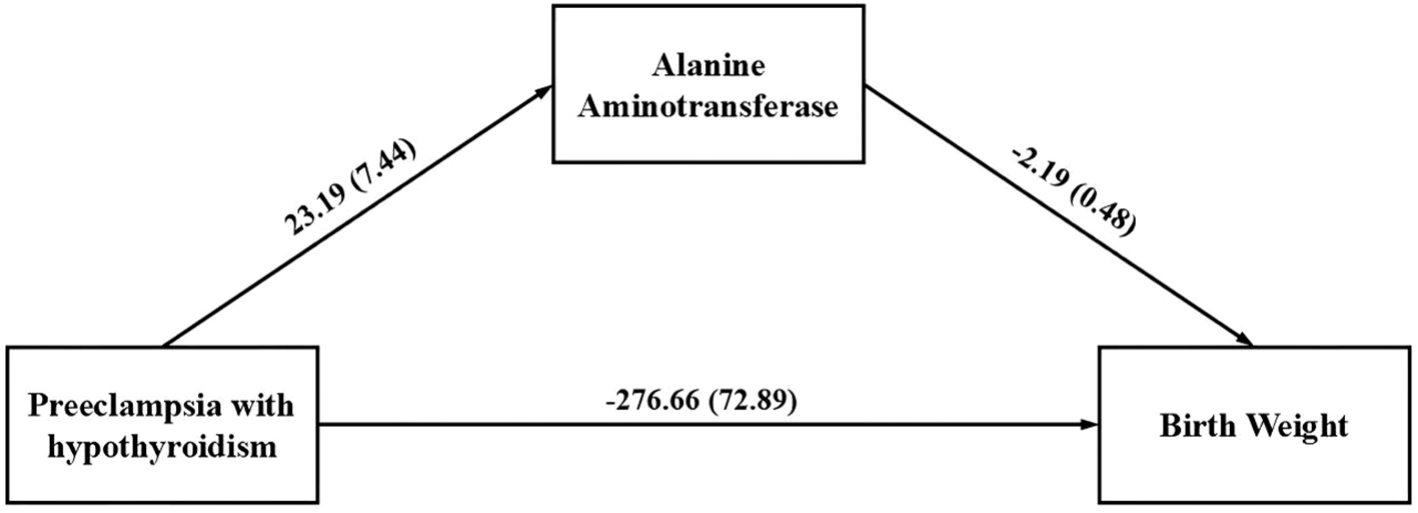
The mediation model examines the indirect correlation between gestational hypothyroidism in pregnant women with preeclampsia and neonatal birth weight through maternal alanine aminotransferase level.

Since there was no statistically significant difference in ALP levels between the preeclampsia group and the preeclampsia combined with GHT group, this study only included ALT, which was statistically significant in the multifactorial analysis, into a mediation model to investigate whether the factor partially mediate the relationship between preeclampsia in pregnant women with GHT and neonatal birth weight. The path coefficients are detailed in **Table 5**. The results indicate that pregnant women with preeclampsia combined with GHT exhibited higher ALT levels compared to those with preeclampsia alone (*β’*=23.19, *P*=0.002), which was associated with a negative impact on neonatal birth weight (*β’*=-271.18, *P*<0.001). Furthermore, ALT levels had a negative effect on neonatal birth weight (*β’*=-2.40, *P*<0.001. In light of these path results, the study explored the mediating effect of ALT, as detailed in **Table 6**. The findings indicate that preeclampsia in conjunction with GHT may indirectly diminish neonatal birth weight by elevating maternal ALT levels. The mediating effect of ALT is quantified at -50.85 with a 95% Bootstrap confidence interval. The interval (95% *CI*) of [101.07, -15.07] does not encompass zero, indicating a substantial mediating effect that accounts for 15.5% of the total effect. The constructed mediation model is illustrated in **Figure 1**.

**Table 5:** Path-coefficients of the mediating models.

Pathway	Standardized coefficients	Coefficients(*β^’^*)	S.E.	*P*
PE&GHT→ALT	0.49	23.19	7.44	0.002
PE&GHT→BW	-0.42	-271.18	72.25	<0.001
ALT→BW	-0.18	-2.40	0.48	<0.001

Adjusting for preterm delivery, fetal growth restriction and pre-pregnancy body mass index.

S.E., Standard Error; PE&GHT, Preeclampsia combined with gestational hypothyroidism; ALT, Alanine Aminotransferase; BW, Birth Weight.

**Table 6:** Mediating effects of maternal alanine aminotransferase between preeclampsia with gestational hypothyroidism and birth weight.

Variable	Estimate	S.E.	Bootstrap 95%*CI*		Effect size (%)
			Lower	Upper	
Total effect	-327.52	73.79	-472.56	-182.48	100.0
Direct effect	-276.66	72.89	-419.94	-133.39	84.5
Indirect effect	-50.85	22.53	-101.07	-15.07	15.5

Adjusting for preterm delivery, fetal growth restriction and pre-pregnancy body mass index.

S.E., Standard Error; CI, Confidence Interval.

## Discussions

This study explores the impact of gestational hypothyroidism and liver function indicators on neonatal birth weight in women with preeclampsia by constructing generalized linear models and mediation models, while also evaluates the potential mediating effects of liver function indicators. The results indicate that approximately 11.0% of the preeclamptic participants included in the study concurrently suffered from hypothyroidism. Neonates born to mothers with preeclampsia and hypothyroidism exhibited lower birth weights; specifically, higher levels of ALT in liver function indicators were associated with lower neonatal birth weights, whereas neonatal birth weight increased with rising ALP levels. Notably, after adjusting for covariates such as preterm birth, FGR, and PBMI, the relationship between AST levels and neonatal birth weight became statistically insignificant. Furthermore, the mediation model revealed that hypothyroidism can directly affect the neonatal birth weight of women with preeclampsia and can also indirectly influence neonatal birth weight through elevated ALT levels (mediating effect: -50.85; 95% *CI* = -101.07, -15.07).

Preeclampsia and gestational hypothyroidism are two common pregnancy complications that can have severe implications for the health of both the mother and the fetus, including miscarriage, preterm birth, fetal growth restriction, and low birth weight (32, 33). Preeclampsia can lead to maternal vascular constriction and reduced blood flow, thereby affecting the blood supply to the placenta and subsequently influencing the nutritional supply to the fetus (34). Particularly, preeclampsia is one of the significant causes of maternal and neonatal mortality, and once diagnosed, there are currently no effective treatment options available aside from the termination of pregnancy (34, 35). Similarly, during pregnancy, thyroid hormones can regulate various metabolic balances in pregnant women and are also involved in the formation and function of the placenta. In cases of hypothyroidism, the resulting deficiency of thyroid hormones may lead to placental dysfunction, causing fetal developmental abnormalities (36). It is worth noting that these two diseases often coexist and influence each other. Previous studies have indicated that hypothyroidism is significantly associated with an increased incidence of preeclampsia (37), and the prevalence of hypothyroidism among patients with preeclampsia is significantly higher than that in the general population (38). Additionally, further research has pointed out that hypothyroidism is correlated with the severity of preeclampsia (39). Therefore, the combination of preeclampsia and gestational hypothyroidism may pose greater risks to both the mother and the fetus. On the other hand, the birth weight of neonates is not only related to their survival rates but also has lasting implications for their physical growth, the development of various systems, and health issues in adulthood (40, 41). That’s why we focused on investigating the effect of hypothyroidism in pregnant women with preeclampsia on neonatal birth weight. Our study findings indicate that neonates born to mothers with preeclampsia combined with hypothyroidism have lower birth weights compared to those born to mothers with preeclampsia alone, which is consistent with previous research results (42). This may indicate that when pregnant women experience preeclampsia in conjunction with hypothyroidism, it may have a more severe impact on the birth weight of the neonate.

Furthermore, our study further investigated the association between maternal liver function indicators and neonatal birth weight. Among pregnant women, the prevalence of liver diseases during pregnancy is approximately 3%, primarily manifested by abnormal changes in transaminases, bilirubin, and other related parameters (43). Pregnancy-related liver diseases are closely associated with fetal growth and development. Some pregnancy-specific liver diseases, such as acute fatty liver of pregnancy (AFLP) and intrahepatic cholestasis of pregnancy (ICP), may result in maternal hepatic dysfunction, which can subsequently affect the placenta’s ability to supply nutrients and oxygen to the fetus. This may lead to complications such as fetal intrauterine distress, preterm birth, and LBW, thereby posing risks to maternal and fetal safety (44). However, during pregnancy, the indicators related to liver function diagnosis do not change independently and may undergo physiological changes, which complicates the diagnosis of liver function in pregnant women. For instance, ALP levels may physiologically increase in the late stages of pregnancy due to placental production and fetal skeletal development. Conversely, albumin levels may decrease due to hemodilution. Nevertheless, when maternal transaminase and bilirubin levels increase, it is generally considered an abnormal phenomenon (45). Consistent with the findings of Sciarrone et, al (46), this study also indicates that maternal ALT levels are negatively correlated with neonatal birth weight. Elevated ALT levels typically indicate liver cell damage or liver dysfunction, which may lead to a decrease in the liver’s synthetic capacity and subsequently affect fetal nutrition supply (47). Additionally, pro-inflammatory factors released due to liver damage can cross the placental barrier and inhibit fetal growth (48), ultimately resulting in reduced the neonatal birth weight (49). This study also found a positive correlation between maternal serum ALP levels and neonatal birth weight, which is consistent with previous research findings (50, 51). The variation in ALP levels is associated with gestational age; although elevated ALP levels are related to ICP (52), which may impair placental function, the increase in ALP during pregnancy is generally considered physiological. From another perspective, ALP is involved in the transport and metabolic processes within the placenta. Elevated ALP levels may reflect robust placental function, which is beneficial for fetal growth and development, thereby contributing to increased birth weight (53). Interestingly, after adjusting for covariates, the statistical significance between AST levels and neonatal birth weight dissipated. This may be attributed to the substantial influence of these covariates on neonatal birth weight, thereby obscuring the effect of AST levels. Additionally, there may be a high correlation between AST levels and the covariates, as indicated by the research conducted by Zhuang et al., which suggests that AST is an independent risk factor for preterm birth (54).

It is noteworthy that both preeclampsia and hypothyroidism can impair maternal liver function (35, 55), and that preeclampsia, hypothyroidism, and liver function all have an impact on neonatal birth weight. Therefore, we constructed a mediation model to explore whether preeclampsia combined with hypothyroidism could indirectly influence neonatal birth weight by altering liver function indicators. This investigation serves as an extension of our understanding of the impact of hypothyroidism on fetal development. The results of this study indicate that ALT levels partially mediate the relationship between preeclampsia combined with hypothyroidism and neonatal birth weight, that is, compared to pregnant women with preeclampsia alone, those with preeclampsia combined with hypothyroidism not only directly contribute to a reduction in neonatal birth weight but may also indirectly lower birth weight through increased ALT levels. Hypothyroidism is characterized by elevated serum TSH levels and decreased FT4 levels, leading to thyroid hormone deficiency, which plays a crucial role in hepatic cellular activity and liver metabolism. Thus, hypothyroidism may lead to hepatic dysfunction, commonly manifested as impaired lipid metabolism (56) and hepatic steatosis (57). Previous studies have primarily focused on the lipid metabolism of pregnant women with hypothyroidism and its impact on pregnancy outcomes (58). However, there is a paucity of research examining the effects of changes in liver function indicators caused by hypothyroidism on pregnancy outcomes. In fact, hypothyroidism can lead to abnormalities in serum liver enzymes. A study analyzing serum data from 10292 outpatient adults indicated a negative correlation between serum GGT and ALT concentrations and FT4 levels (59), suggesting that hypothyroidism may result in elevated concentrations of ALT and gamma-glutamyl transferase (GGT).

Certainly, this study has several limitations. Firstly, the scope of our investigation is not comprehensive enough, as it lacks details on factors such as the nutritional status of pregnant women and the treatment received during hospitalization, which may confound the relationship with neonatal birth weight. Secondly, given that this study employs an observational design, it cannot establish causal relationships, necessitating cautious interpretation of the findings. Thirdly, the study only included women with singleton live births, which may introduce selection bias; additionally, there is a lack of relevant data from normal pregnant women to serve as a control group. Fourthly, since the focus of research designs was on examining the effects of preeclampsia combined with or without hypothyroidism on maternal liver function and neonatal birth weight, the impact of the severity of preeclampsia on liver function and birth weight was overlooked. Consequently, some classification criteria lacked comprehensive data monitoring, making it impossible to conduct more in-depth exploratory research. Finally, there may exist a bidirectional relationship between thyroid function and liver function; this study only explored whether hypothyroidism could induce changes in liver function indicators that indirectly affect neonatal birth weight. Therefore, future research should consider conducting larger-scale prospective studies to gain a more comprehensive understanding of the intricate interplay between preeclampsia with hypothyroidism, liver function, and adverse pregnancy outcomes.

## Conclusions

This study provides new insights by exploring the impact of hypothyroidism and liver function indicators in pregnant women with preeclampsia on neonatal birth weight. The findings support the notion that hypothyroidism adversely affects fetal development and suggest that maternal serum ALT levels may serve as a potential partial mediator linking preeclampsia combined with hypothyroidism and neonatal birth weight. Clinicians should closely monitor thyroid and liver function in pregnant women with preeclampsia and implement appropriate interventions to improve neonatal birth weight and health outcomes.

## Data Availability

The raw data supporting the conclusions of this article will be made available by the authors, without undue reservation.
