# Supplementary material for: The association between gestational hypothyroidism in pregnant women with preeclampsia, maternal liver function indicators, and neonatal birth weight: a study in Chinese pregnant women
Source: Front Endocrinol (Lausanne). 2025 Sep 22;16:1555277. doi: 10.3389/fendo.2025.1555277 (PMC12498002; doi:10.3389/fendo.2025.1555277)
Supplement: Supplementary file 1 [file Table1.docx]

**Table S1** **Comparison of maternal characteristics by neonatal birth weight status**

| Variable | neonatal birth weight status [N (%)] | | $\chi^{2}$ | *P* |
| --- | --- | --- | --- | --- |
|  | non-LBW | LBW |  |  |
| **Age (Years)** |  |  |  |  |
| <25 | 21(91.3) | 2(8.7) | 6.52 | 0.089 |
| 25~35 | 238(78.5) | 65(21.5) |  |  |
| 35~40 | 54(68.4) | 25(31.6) |  |  |
| ≥40 | 12(80.0) | 3(20.0) |  |  |
| **Fetal sex** |  |  |  |  |
| Male | 177(79.4) | 46(20.6) | 1.08 | 0.299 |
| Female | 148(75.1) | 49(24.9) |  |  |
| **Ethnicity** |  |  |  |  |
| Han ethnicity | 320(77.9) | 91(22.1) | 2.50 | 0.114 |
| Ethnic minorities | 5(55.6) | 4(44.4) |  |  |
| **Residence** |  |  |  |  |
| Urban area | 271(77.7) | 78(22.3) | 0.09 | 0.770 |
| Rural area | 54(76.1) | 17(23.9) |  |  |
| **Education level** |  |  |  |  |
| Junior high school and below | 29(70.7) | 12(29.3) | 2.29 | 0.515 |
| High school and vocational secondary school | 37(72.5) | 14(27.5) |  |  |
| Junior college | 107(79.9) | 27(20.1) |  |  |
| Undergraduate and postgraduate degrees | 152(78.4) | 42(21.6) |  |  |
| **PTD** |  |  |  |  |
| No | 294(95.1) | 15(4.9) | 210.81 | <0.001 |
| Yes | 31(27.9) | 80(72.1) |  |  |
| **History of cesarean section** |  |  |  |  |
| No | 248(77.7) | 71(22.3) | 0.10 | 0.753 |
| Yes | 77(76.2) | 24(23.8) |  |  |
| **History of adverse obstetric** |  |  |  |  |
| No | 286(78.8) | 77(21.2) | 3.03 | 0.082 |
| Yes | 39(68.4) | 18(31.6) |  |  |
| **Primipara** |  |  |  |  |
| No | 125(74.9) | 42(25.1) | 1.01 | 0.314 |
| Yes | 200(79.1) | 53(20.9) |  |  |
| **FGR** |  |  |  |  |
| No | 321(81.1) | 75(18.9) | 53.61 | <0.001 |
| Yes | 4(16.7) | 20(83.3) |  |  |
| **Family history of hypertension** |  |  |  |  |
| No | 287(77.6) | 83(22.4) | 0.06 | 0.804 |
| Yes | 38(76.0) | 12(24.0) |  |  |
| **PBMI (kg/m^2^)** |  |  |  |  |
| <18.5 | 11(73.3) | 4(26.7) | 11.51 | 0.009^*^ |
| 18.5~24.9 | 165(71.7) | 65(28.3) |  |  |
| 25.0~29.9 | 102(82.9) | 21(17.1) |  |  |
| ≥30 | 47(90.4) | 5(9.6) |  |  |
| **PE&GHT** |  |  |  |  |
| No | 302(80.7) | 72(19.3) | 22.13 | <0.001 |
| Yes | 23(50.0) | 23(50.0) |  |  |
| **ALT** |  |  |  |  |
| normal | 307(82.5) | 65(17.5) | 49.25 | <0.001 |
| abnormal | 18(37.5) | 30(62.5) |  |  |
| **AST** |  |  |  |  |
| normal | 292(83.0) | 60(17.0) | 38.59 | <0.001 |
| abnormal | 33(48.5) | 35(51.5) |  |  |
| **ALP** |  |  |  |  |
| normal | 303(77.9) | 86(22.1) | 0.79 | 0.375 |
| abnormal | 22(71.0) | 9(29.0) |  |  |
| **TP** |  |  |  |  |
| normal | 236(82.8) | 49(17.2) | 14.92 | <0.001 |
| abnormal | 89(65.9) | 46(34.1) |  |  |
| **Alb** |  |  |  |  |
| normal | 226(83.1) | 46(16.9) | 14.37 | <0.001 |
| abnormal | 99(66.9) | 49(33.1) |  |  |
| **TBIL** |  |  |  |  |
| normal | 316(78.2) | 88(21.8) | 4.24 | 0.039* |
| abnormal | 9(56.3) | 7(43.8) |  |  |

**P*<0.05; Abbreviations: LBW, Low birth weight; PTD, Preterm delivery; FGR, Fetal growth restriction; PBMI, Pre-pregnancy body mass index; PE&GHT: Preeclampsia combined with gestational hypothyroidism; ALT, Alanine Aminotransferase; AST, Aspartate Aminotransferase; ALP, Alkaline Phosphatase; TP, Total Protein; Alb, Albumin; TBIL, Total Bilirubin; BW, Birth Weight.

**Table S2** Logistic regression analysis of risk factors for low birth weight

| Variable | Model 1 | |  | Model 2 | |
| --- | --- | --- | --- | --- | --- |
|  | *OR* (95%*CI*) | *P* |  | *OR*(95%*CI)* | *P* |
| **PE&GHT** |  |  |  |  |  |
| No (ref.) | — | — |  | — | — |
| Yes | 2.73(1.32,5.64) | 0.007 |  | 6.47(1.96,21.34) | 0.002 |
| **ALT** |  |  |  |  |  |
| normal(ref.) | — | — |  | — | — |
| abnormal | 4.66(1.82,11.94) | 0.001 |  | 10.40(2.11,51.35) | 0.004 |
| **AST** |  |  |  |  |  |
| normal(ref.) | — | — |  | — | — |
| abnormal | 1.44(0.62,3.33) | 0.394 |  | 0.87(0.21,3.54) | 0.843 |
| **TP** |  |  |  |  |  |
| normal(ref.) | — | — |  | — | — |
| abnormal | 1.57(0.81,3.02) | 0.180 |  | 1.349（0.49,3.72） | 0.570 |
| **ALB** |  |  |  |  |  |
| normal(ref.) | — | — |  | — | — |
| abnormal | 1.83(0.95,3.55) | 0.073 |  | 2.21(0.79,6.16) | 0.129 |
| **PTD** |  |  |  |  |  |
| No (ref.) | — | — |  | — | — |
| Yes |  |  |  | 112.93(42.20,302.22) | <0.001 |
| **FGR** |  |  |  |  |  |
| No (ref.) | — | — |  | — | — |
| Yes |  |  |  | 26.65(4.62,153.70) | <0.001 |
| **PBMI (kg/m^2^)** |  |  |  |  |  |
| <18.5 |  |  |  | 0.78(0.09,6.76) | 0.818 |
| 18.5~24.9 (ref.) | — | — |  | — | — |
| 25.0~29.9 |  |  |  | 0.35(0.13,0.90) | 0.030 |
| ≥30.0 |  |  |  | 0.10(0.03,0.38) | 0.001 |

** P*<0.05. Abbreviations: CI, confidence interval; PE&GHT: Preeclampsia combined with gestational hypothyroidism; ALT, Alanine Aminotransferase; AST, Aspartate Aminotransferase; TP, Total Protein; Alb, Albumin; PTD, Preterm delivery; FGR, Fetal growth restriction; PBMI, Pre-pregnancy body mass index; ref., Reference Group.

Model 1 was the unadjusted model; Model 2 adjusted for PTD, FGR and PBMI.
